# The Effect of Egg Yolk Oil in the Healing of Third Degree Burn Wound in Rats

**Published:** 2011-10-01

**Authors:** F Rastegar, N Azarpira, M Amiri, A Azarpira

**Affiliations:** 1 Organ Transplant Research Center, Nemazee hospital, Shiraz University of Medical Sciences, Shiraz, Iran; 2 Great Lakes Bioenergy Research Center, University of Wisconsin, Madison, Madison, USA

**Keywords:** Burn, Egg yolk, Wound, Silver sulfadiazine, Rat

## Abstract

**Background:**

Burn injury is a major cause of death and disability worldwide. A domestic medication in wound healing, preventing infection and reduction of scar tissue as well as availability is still an important challenge. This study aims to evaluate the efficacy of yolk egg oil in treatment of burn wounds in rats.

**Methods:**

A standard 3rd degree burn wound was produced and the animals were divided into three groups according to topical treatment including yolk oil, 1% silver sulfadiazine (SSD) and control. In days 7, 14 and 30,animal’s weight, wound size, as well as histopathological findings of skin were evaluated in different groups.

**Results:**

Average size of wound after 7 days was 3.4, 5.3, and 6.7 cm in yolk oil, SSD and control groups. There were significant differences between yolk oil and the other groups in this aspect. The wound size of yolk egg group was also significantly smaller than other groups in 14th and 30th days. Results of the histological studies indicated significant differences between yolk oil, SSD and control groups on day 30, with mean score of 3.75, 3.5 and 2.8 respectively. The difference between yolk oil and the other groups was significant.

**Conclusion:**

Yolk oil–treated burned animals showed abundant re-epithelialization without tissue scar in comparison with SSD group. Although the egg yolk has many vital nutrients, but its exact mechanism in healing process is unknown. Therefore, further studies evaluating the influence of individual components on burn-healing process is advised.

## Introduction

Million peoples annually suffer from burn injuries and it is a major cause of death and disability in the world. Therefore, a need for a local and effective wound healing medication and in preventing infection, decreasing fluid imbalance, promoting re-epithelialization, reducing the occurrence of scar tissue and a good availability is an important challenge. Silver sulfadiazine (1% SSD) cream is the most common used topical treatment for treatment of burn injury.[1] Its potent anti-microbial efficacy is the main reason for widespread usage.[2][3][4] However, it has systemic complications such as neutropenia, renal toxicity and methaemoglobinemia.[5][6] Delayed wound healing following treatment is another unfavorable clinical adverse effect of topical silver ointments.[3] There are some studies on animal models reporting traditional and domestic remedies in burns.[7][8][9]

The oil of egg made from the yolk consists largely of a high fat, protein, carbohydrate, ash and water that seem excellent in healing process. Avian eggs are preferable in view of their low cost and their ready availability from chickens and turkeys. Various portions of the avian egg were found useful in pharmaceutical and cosmetic preparations. The albumin or white portion of the egg has been utilized in a pliable bacteriostatic coagulum for the treatment of burns as described in literature.[10] In this prospective randomized trial, wounds were treated with oil prepared from yolk egg and SSD and the results were compared histologically to assess the rate of wound healing.

## Materials and Methods

Fresh farm eggs were purchased from a local grocery store. Egg shell was carefully broken, the yolk was separated from the egg white, and the egg yolk oil was extracted as described by Warren M. et al.[11]

Sixty male Wistar rats weighing 180 to 200 grams were used and housed under standard conditions. All experiments, care and the sacrifice procedure were under supervision of Animal Care Committee of Iran Veterinary Organization. The rats were sedated by intramuscular injection of ketamine and xylocain. The back hairs were shaved and a standard 3rd degree burn wound was produced using a hot plate with the same size about 20% total body surface area as previously described.[12] After 24 hours, the animals were randomly divided into three groups (n=20/group). Group 1 was treated with oil made from the yolk of egg, group 2 received 1% SSD (Cina Daru Company, Tehran, Iran) and group 3 was the control group getting no topical agent. The wounds were treated twice daily. They were observed daily for evidence of infection, exudates or tightening of the skin in the burn area.

In order to quantify the rate of wound healing, the weight of animals and the size of lesions were determined at 7, 14 and 21 days after burn injury. On days 7, 14 and 21 of treatment, the animals were sacrificed with an overdose of anesthetics; the burn areas were removed and fixed in formalin. Tissue sections were prepared and stained with hematoxylin and eosin (H and E). Histological scores were expressed as described by Galeano et al.,[13] including degree of epithelialization (absence or presence of epithelial covering, crusting, spongiosis and intraepithelial inflammatory cells), granulation tissue and collagen matrix organization (adipose tissue substitution as an index of impaired wound closure, dense eosinophilic collagen matrix, edema, hemorrhage, degree of inflammation, number and organization of fibroblasts with shapes such as a plumped, spindle, or stellate-morphology), inflammatory infiltrates (neutrophile and lymphocytes), and angiogenesis (number of capillary lumens, congestion, fibrin deposition, hemorrhage). The histological scoring system ranged between 1 and 4 as described previously.[13]

The results were expressed as mean±SD. The data were analyzed using SPSS software (version 11.5, Chicago, IL, USA) by non-parametric tests of Kruskal-Wallis and Mann-Whiteney tests. A p<0.05 was considered statistically significance.

## Results

Average weight of animals in three groups of yolk oil, silver sulfadiazine and control before beginning of this study was 185±23.6 grams, and after 7 days of induction of burn decreased to 198, 164 and 155 grams respectively. There were significant differences between groups in the reduction of weight. The average weight of animal in all groups increased after the treatment period (21 days, Table 1). There were significant differences between yolk oil and the other groups in this aspect.

**Table 1:** Body weights of treated rats with yolk egg, silver sulfadiazine (SDD) and control in different days of experiment.

**Groups**	**Days after burn injury**		
	** 7 **	** 14**	** 30**
Yolk egg (g)	198.3±10^a^	235.3±11^b^	267.1±11^c^
SDD (g)	164.9±12	200.5±10	222.6±9
Control (g)	155.5±9	207.3±8	238.8±10

^a^ P-value=0.012

^b^ P-value= 0.015

^c^ P-value=0.018

Skin lesions were measured on 7, 14 and 21 days after burn injury. Average size of wound after 7 days was 3.4, 5.3, and 6.7 Cm in yolk oil, SSD and control groups (Table 2). There were significant differences between yolk oil and the other groups in this aspect. There was also statistical difference among yolk oil and the others in wound size on the 30th day of treatment (p=0.009).

**Table 2:** Average wound size of treated animals with yolk egg, silver sulfadiazine (SDD) and control in different days of experiment

**Groups**	**Days after burn injury**		
	**7**	**14**	**30**
Yolk egg (cm)	3.43± 0.5 a	1.44±0.3^a^	0.09±0.4^a^
SDD (cm)	5.37±0.4	2.63± 0.5	1.32±0.7
Control (cm)	6.75±0.5	3.55±0.6	1.8±1.7

^a^ P-value<0.001

**Fig. 1:**
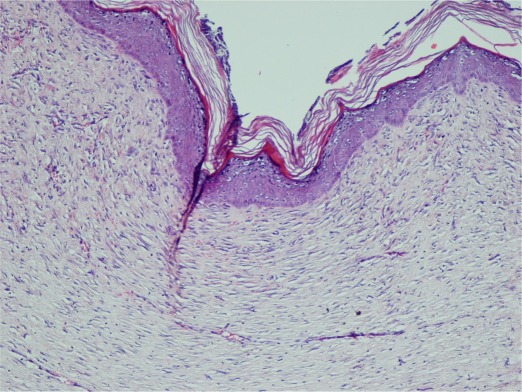
Complete re-epithelialization, low inflammatory cell infiltrate in a well- formed granulation tissue (H and E ×100).

Results of the histological studies indicated significant differences between yolk oil, SSD and control groups on day 30, with mean score of 3.75, 3.5 and 2.8 respectively (Table 3). Yolk egg group exhibited good re-epithelialization with no evidence of crusting or scar formation (Figure 1). Granulation tissue formation consisted of oval or spindle fibroblasts embedded in eosinophilic collagen matrix and oriented parallel to the epithelial surface were noticed. There were few inflammatory cell infiltrations, mainly in perivascular site. The number of new capillaries within the granulation tissue was significantly higher. Minimal interstitial edema with no evidence of hemorrhage was also identified.

**Table 3:** Histological scores in burn wounds from rats treated with yolk egg, silver sulfadiazine (SDD) and control.

**Groups**	**Days after burn injury**		
	**7**	**14**	**30**
Yolk egg	0.9± 0.1 a	3.5±1.3 a	3.75±0.25 a
SDD	0	2.45± 0.75	3.5±0.5
Control	0	1.35±0.7	2.8±0.2

^a^ P-value<0.001

**Fig. 2:**
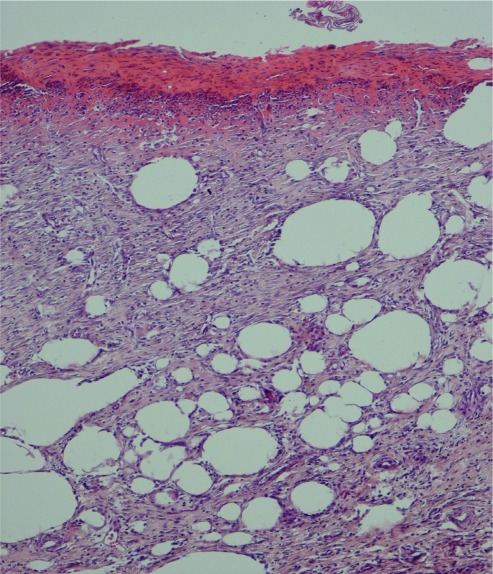
Lack of re-epithelialization with immature granulation tissue formation as well as adipose tissue substitution in control group (H and E ×100).

The control group showed loose collagen matrix with scattered plumped fibroblasts with edema, hemorrhage, and fibrin deposition. Granulation tissue with few congested capillary was seen. The dermal layer was occupied by adipose tissue as indexes of immaturity (Figure 2). In epidermis, absence of epithelial covering with evidence of crusting or incomplete monolayer of epidermal cells was observed.

## Discussion

An egg yolk is the part of an egg that serves as the food source for the developing embryo inside. All of the egg's fat and cholesterol, and almost half of the protein are present in egg yolk. Lipids of yolk are exclusively associated with lipoprotein assemblies. They are made up of 62% triglycerides, 33% phospholipids, and less than 5% cholesterol. The omega 3 fatty acids, mainly found in phospholipids, are another important component of yolk, which is considered an essential nutrient for brain function and visual acuity in humans.[7][14][15] The composition of the fatty acids in egg yolk included unsaturated fatty acids of oleic acid, linoleic acid, palmitoleic acid, linolenic acid and saturated fatty acids consisting of palmitic and stearic acid.

All of the fat soluble vitamins are found in the egg yolk as one of the few foods naturally containing vitamin D. Carotenoids are the natural pigments of hen egg yolk that confer with its yellow color. Egg yolk also contains immunoglobulin yolk (IgY). It is transferred from the laying hen to the egg yolk by passive immunity to protect both embryo and hatchling from microorganism invasion. It is supposed that it has antioxidant properties.[14][15] According to above mentioned points, egg yolk has many vital nutrients that are used in medical, pharmaceutical and cosmetic industries.

Thermal burn injury is still a major cause of death and disability in the world and its healing process is a challenge in modern medicine. Burn in human body may be treated by different methods depending on the extent and severity of the burn.[16] SSD is commonly used to prevent and treat infections of the second and third-degree, burns. It kills a wide variety of bacteria.[17][18]

Epidermal regeneration of a wound is a complex process that the residual epithelial cells proliferate in an integrated manner to form an intact epidermis.[19][20] In this study, yolk oil –treated burned animals showed abundant re-epithelialization without any tissue scar in comparison with SSD group. It is suggested that biological materials of the yolk are responsible for the reduction in catabolism as well as increased matrix synthesis and promotion of re-epithelialization. However, further studies evaluating the influence of individual components with different dosage on maturation and proliferation of cellular components would provide insights into mechanism of accelerated wound healing.
